# Supplementary material for: Venomix: a simple bioinformatic pipeline for identifying and characterizing toxin gene candidates from transcriptomic data
Source: PeerJ. 2018 Jul 31;6:e5361. doi: 10.7717/peerj.5361 (PMC6074769; doi:10.7717/peerj.5361)
Supplement: Supplemental Information 3 [file peerj-06-5361-s003.gz › FinalOutput_E-6/Snaclec_lebecetin_subunit_alpha_3/finaltree.pdf]

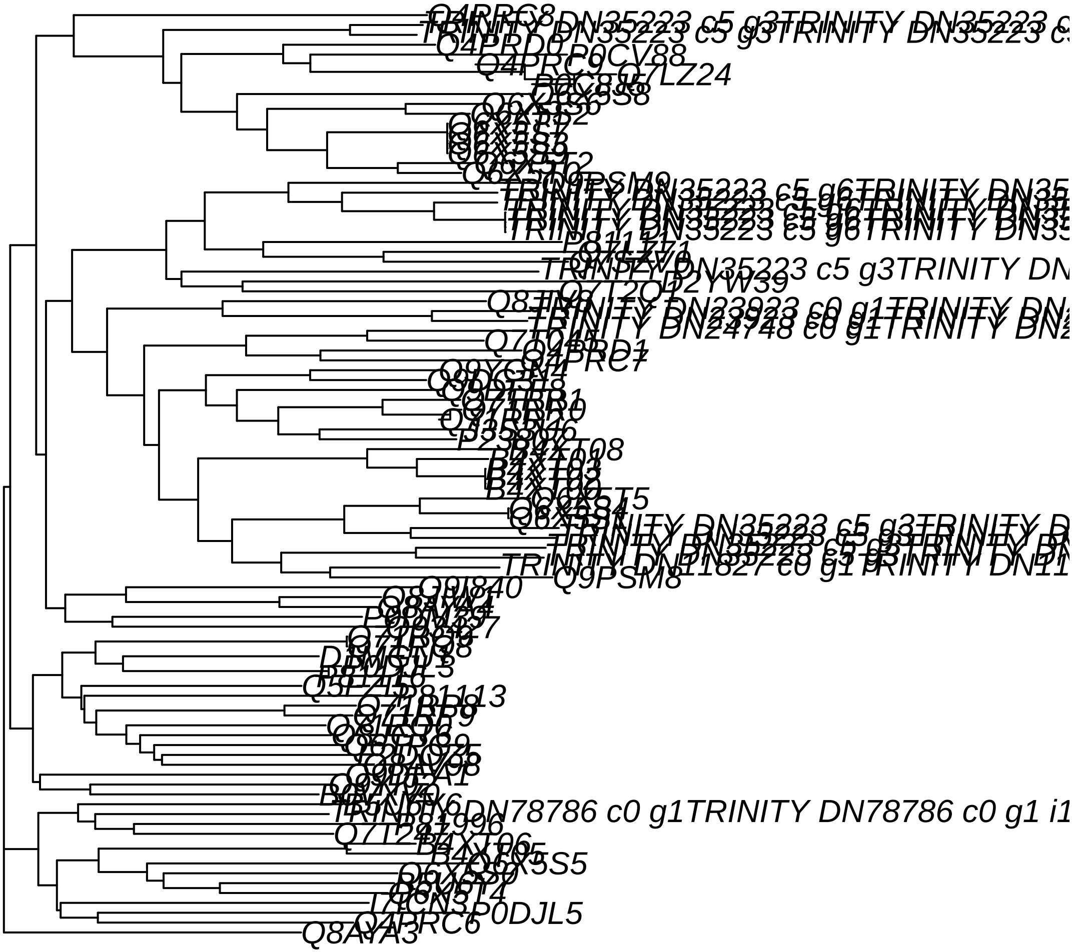

Q4PRC8
P0CV88
Q7LZ24
D2YW39
Q7T045
Q4PRC7
B4XT08
Q9PSM8
Q9I840
TRINITY DN78786 c0 g1TRINITY DN78786 c0 g1 i1
B4XT06
Q6X5S5
P0DJL5
Q4PRC6
Q8AYA3
